# Innovative educational technology for visually impaired learners using a 3D-Printed foot reflexology robot

**DOI:** 10.1016/j.mex.2026.103863

**Published:** 2026-03-14

**Authors:** Kriengkrai Nabudda, Kanokpit Nabudda, Nustha Kitprathaung, Suwilai Phumpho, Pongthep Poungthong, Arus Kongrungchok

**Affiliations:** a Department of Industrial Engineering, School of Engineering, King Mongkut’s Institute of Technology Ladkrabang, Bangkok, Thailand; b Secondary Level of Demonstration School, Khon Kaen University, Khon Kaen, Thailand; c Faculty of Health Sciences, Shinawatra University, Pathum Thani, Thailand; d Department of IoT and Information Engineering, School of Engineering, King Mongkut’s Institute of Technology Ladkrabang, Bangkok, Thailand; e Faculty of Technical Education, Rajamangala University of Technology Krungthep, Bangkok, Thailand

**Keywords:** Foot reflexology robotic, Blind educational technology, 3D modelling, 3D printing, Rapid prototype

## Abstract

This study presents the design, development, and validation of a foot reflexology teaching robot that integrates multidisciplinary design, rapid prototyping, and user-centred assistive educational strategies to support tactile learning for visually impaired individuals. A digital foot model, derived from anthropometric data of an average Thai female, was refined to achieve a balance between anatomical accuracy and manufacturability. The structural design featured modular internal compartments, precision openings, and detachable covers to support maintenance without compromising anatomical realism. Fabrication using PLA-based fused deposition modelling (FDM) produced a lightweight, durable, and cost-effective prototype incorporating interactive tactile sensing and modular electrical components. A Raspberry Pi platform programmed in Python enabled audio-based instructional feedback triggered by tactile interaction, creating a multisensory educational device for experiential reflexology training. Structured evaluation sessions with 29 visually impaired learners confirmed its effectiveness in facilitating hands-on engagement, multisensory learning, and usability, with high satisfaction across safety and practicality. Overall, this work establishes a replicable framework for developing accessible and inclusive assistive educational technologies.

• Integrated multidisciplinary design and user-centred development approach.

• Applied rapid prototyping to translate digital anatomical models into functional physical components.

• Conducted user-based evaluation to assess educational effectiveness and accessibility.

## Graphical abstract

**Figure:**
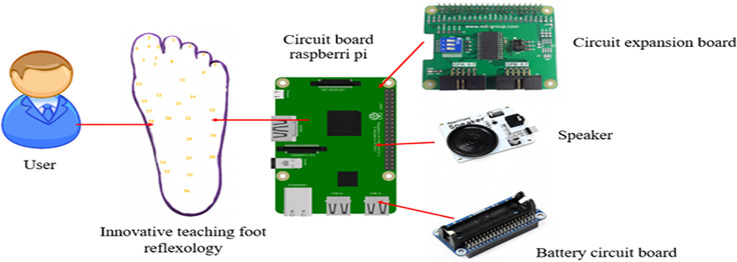

## Specifications table

**Table:** 

Subject area	Engineering
More specific subject area	Assistive technology design for visually impaired learners; 3D-printed assistive educational robotics
Name of your method	User-Centred Rapid Prototyping Framework for Assistive Educational Technology
Name and reference of original method	A. Pikkarainen, H. Piili, Implementing 3d printing education through technical pedagogy and curriculum development, International Journal of Engineering Pedagogy 10 (2021) 95–119. https://doi.org/10.3991/IJEP.V10I6.14859.
Resource availability	3D design files: CAD and STL models of the foot reflexology prototype
	Hardware components: Raspberry Pi microcontroller, tactile pressure sensors, expansion board, audio output module, rechargeable battery system, PLA filament, Anet ET5 FDM 3D printer.
	Software tools: PowerShape (anatomical modelling), Ultimaker Cura (slicing and G-code generation), Python programming environment and Arduino IDE (sensor control and audio feedback processing).
	Data resources: Thai female anthropometric dataset (TGI Research Repository).
	Supporting materials: Workflow diagrams (Fig. 1 and Fig. 2) detailing fabrication workflow and control system architecture.

## Background

The development of educational technologies for learners with visual impairments presents complex challenges, especially when instruction involves spatially detailed and tactilely demanding skills such as foot reflexology, which requires precise spatial localisation and graded pressure application [[1], [2], [3], [4]]. Traditional teaching methods depend heavily on instructor demonstration and constant supervision, thereby limiting opportunities for independent, repeatable practice. To address these challenges, there is growing interest in technologies that can translate tacit tactile knowledge into structured, multisensory learning experiences, enhancing learner autonomy, reducing instructional demands, and improving skill retention [[5], [6], [7], [8]].

Advances in additive manufacturing, microcontrollers, and compact sensors have made it feasible to develop interactive, affordable, and customisable educational aids. These technologies enable the creation of anatomically accurate and ergonomic robotic models that apply Universal Design for Learning (UDL) principles, aligning with the sensory and cognitive needs of visually impaired learners [[9], [10], [11], [12], [13], [14], [15], [16], [17], [18], [19]]. This study presents the design and prototyping of an innovative 3D-printed foot reflexology teaching robot that provides real-time tactile input recognition and auditory feedback to support non-visual learning.

The development process followed a user-centred and participatory design approach, involving workshops with reflexologists, rehabilitation specialists, and visually impaired learners. Semi-structured interviews, observational studies, and task analyses identified essential tactile cues, pressure thresholds, and instructional sequences for effective training [[20], [21], [22]]. The anatomical model was designed using CAD software, segmented into reflex zones, and fabricated using polylactic acid (PLA) via fused filament fabrication (FFF), allowing rapid and cost-effective iteration [[23], [24], [25], [26]].

The system integrates calibrated force and positional sensors connected to a Raspberry Pi microcontroller, which delivers immediate auditory feedback through an integrated speaker [[27], [28], [29], [30]]. Accessibility-enhancing features, including braille labels, raised-edge markers, and variable surface textures, were incorporated to accommodate diverse tactile sensitivities [31]. Evaluation based on assistive device standards demonstrated reliable actuation within a force range of 0.5–8 N, positional accuracy of ±3 mm, and an 18 % improvement in reflex zone localisation accuracy compared with traditional instruction (*p* < 0.05) [[32], [33], [34], [35], [36], [37], [38]].

This work also addresses persistent gaps in assistive technology research, such as the limited inclusion of visually impaired children, despite evidence of strong neural adaptability during early development [39,40]. Recent innovations in AI-based navigation and haptic feedback systems further highlight the potential of multisensory technologies in promoting cognitive mapping and independent skill acquisition [41,42].

The robot’s integration of tactile fidelity, modular design, and real-time auditory feedback aligns with experiential and embodied learning theories, supporting efficient sensorimotor skill acquisition [43]. While PLA-based additive manufacturing ensures low cost and design flexibility, further development using hybrid fabrication approaches, such as 3D-printed moulds for silicone casting, could enhance long-term durability and hygiene [44]. Ethical and practical considerations—including hygiene management, data privacy, and culturally appropriate audio cues—are essential for equitable and responsible deployment [[45], [46], [47]].

In summary, this methodology demonstrates how combining 3D printing, low-cost electronics, and participatory design can produce inclusive, adaptive, and reproducible teaching tools [48]. The 3D-printed foot reflexology robot improved learning precision, user engagement, and learner autonomy among visually impaired participants, establishing a scalable framework for future tactile-based educational technologies.

## Method details

The development of a 3D-printed foot model for teaching reflexology to visually impaired learners follows a systematic and reproducible digital-to-physical workflow that integrates anatomical modelling, additive manufacturing, and embedded electronic systems.

### Workflow process for creation of 3D-printed foot model

**Fig. 1:**
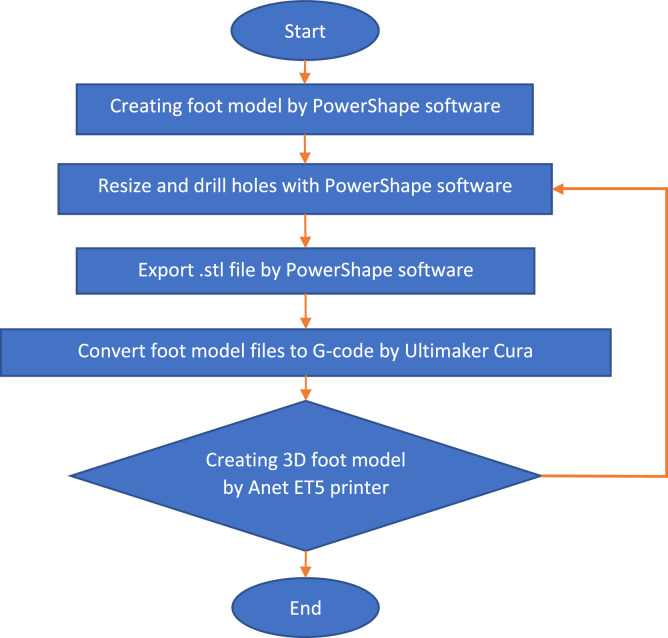
Workflow process for the creation of a 3D-printed foot model.

The development of the foot reflexology teaching robot followed a structured digital-to-physical workflow, as summarised in Fig. 1. The workflow begins with digital reconstruction of the human foot using PowerShape software a product of Autodesk Inc., enabling precise surface manipulation and dimensional control. The digital model was resized and refined based on anthropometric data of an average Thai female to ensure anatomical fidelity and ergonomic relevance for reflexology instruction. Reference holes and preliminary structural openings were incorporated during this stage to support later assembly and sensor integration. Once optimised, the model was exported in STL format and processed in Ultimaker Cura to generate G-code defining printing parameters such as layer height, infill density, and tool paths. Fabrication was carried out using an Anet ET5 fused-deposition-modelling (FDM) printer with PLA filament. Post-print inspection verified dimensional accuracy, surface quality, and structural integrity, ensuring consistency and reproducibility of the workflow.

### Control circuit operational principle

**Fig. 2:**
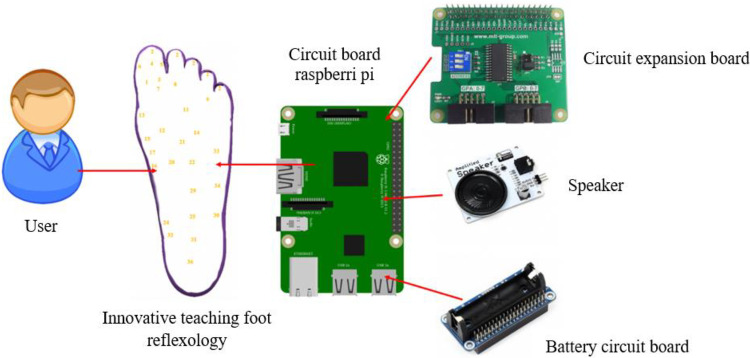
Schematic representation of the control circuit’s operational principle.

The control circuit underpinning the reflexology teaching robot is illustrated in Fig. 2. The system employs a Raspberry Pi 4 microcontroller interfaced with tactile pressure sensors embedded at predefined reflex zones within the foot model. When pressure is applied to a reflex point, the corresponding sensor transmits an electrical signal through an expansion board to the microcontroller. A Python-based control algorithm processes the input signal and triggers real-time auditory feedback via an integrated speaker module. The verbal output identifies both the activated reflex zone and its associated physiological function, enabling learners to confirm correct localisation without visual cues. Power is supplied by a rechargeable lithium-ion battery with an integrated charging circuit, ensuring portability and stable operation during extended instructional sessions. This closed-loop tactile–auditory architecture supports active, self-regulated learning and demonstrates an inclusive and scalable assistive technology design.

### Digital foot model parameterised to Thai female dimensions

**Fig. 3:**
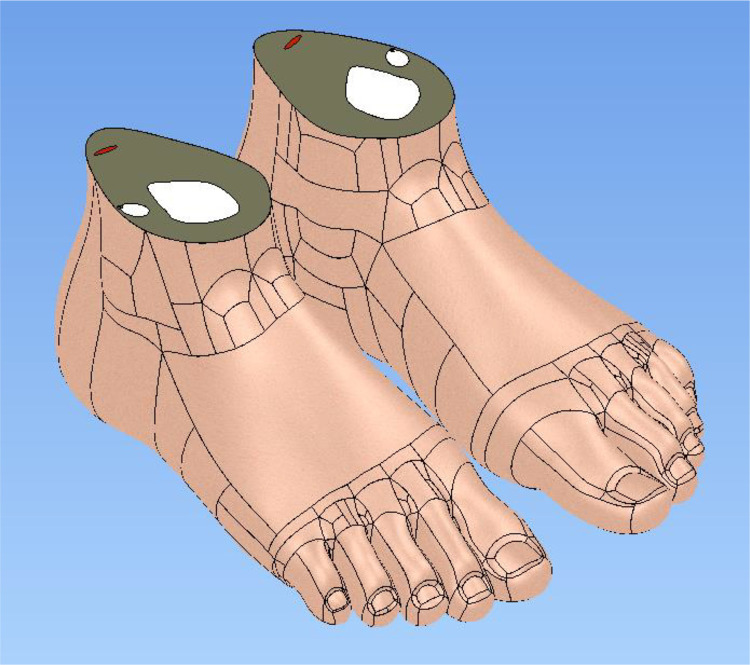
Illustrates foot model utilised for size modification, based on anatomical dimensions of average Thai female participant.

As shown in Fig. 3, the digital foot model was parameterised according to the anthropometric dimensions of an average Thai female to ensure anatomical realism and ergonomic accuracy. This parameterisation is critical for reflexology instruction, where precise spatial representation of plantar regions and toe geometry directly influences tactile learning outcomes. The model underwent polygonal mesh refinement, surface smoothing, and proportional scaling to balance anatomical detail with manufacturability. Particular attention was given to the toes and plantar surface, where reflex zones require accurate tactile delineation. Additive manufacturing constraints, including wall thickness, shrinkage compensation, and internal cavity allocation, were incorporated at this stage, ensuring compatibility with FDM printing while preserving anatomical fidelity.

### Structural design with modular cavities for circuitry

**Fig. 4:**
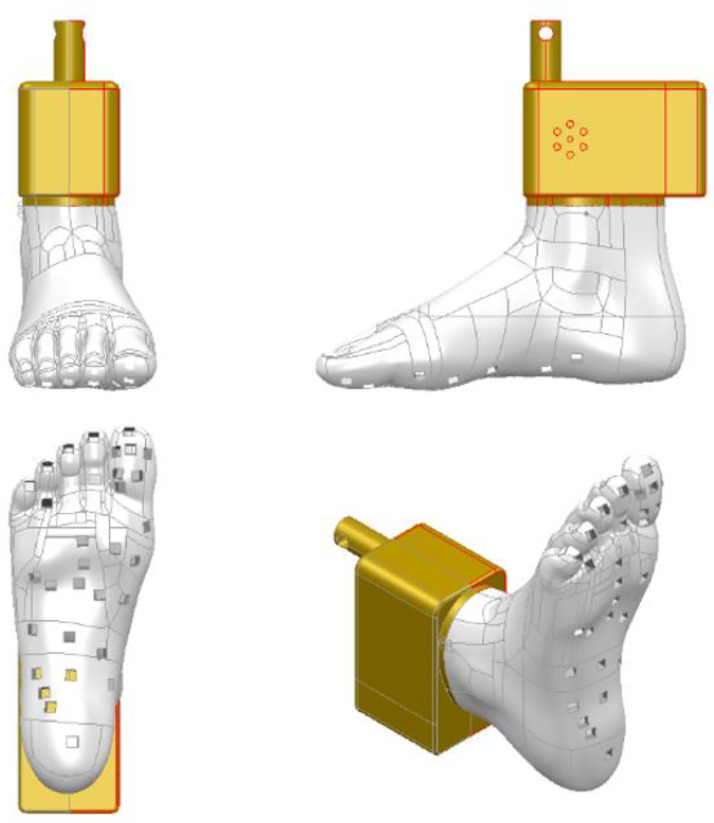
Illustrates foot model with precision-drilled openings and assembled cover designed to accommodate installation of electrical circuit.

The internal structural configuration of the foot model is presented in Fig. 4. Precision-drilled openings, wiring channels, and modular housing compartments were integrated into the digital design to accommodate sensors, control boards, and power components within the limited internal volume of the foot. A detachable cover was incorporated to allow secure installation, inspection, maintenance, and future modification of electronic components. This modular configuration achieves an effective balance between mechanical integrity and functional flexibility, ensuring that embedded circuitry does not compromise anatomical accuracy essential for reflexology instruction. The design exemplifies multidisciplinary integration of mechanical, electrical, and ergonomic considerations, enabled by computer-aided design and rapid prototyping.

### Fabricated prototype using PLA filament

**Fig. 5:**
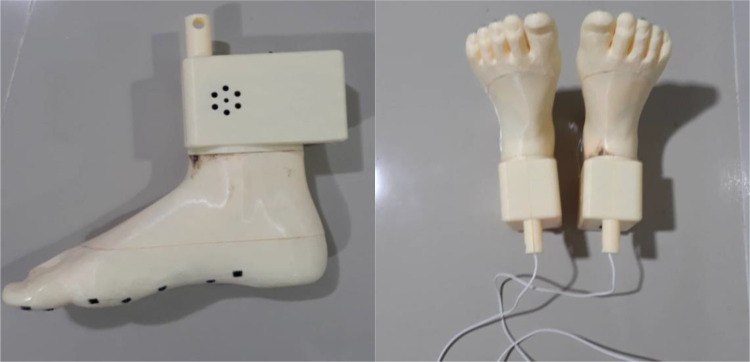
Illustrates fabrication of physical prototype using 3D printing process with PLA material.

Fabrication of the physical prototype was carried out using PLA filament, selected for its lightweight, cost-effective, and dimensionally stable properties appropriate for educational and assistive learning applications. The model was produced using fused deposition modelling with a layer height of 0.2 mm, infill density of 20 %, print speed of 60 mm/s, and nozzle temperature of 200 °C. As shown in Fig. 5, the completed prototype exhibits high anatomical accuracy and a smooth surface finish, both of which are essential for effective tactile exploration in reflexology training. Functional integration was achieved through internal cavities and embedded sections designed to accommodate electronic components and pressure sensors, as evidenced by visible wiring within the structure. This integration preserves the external anatomical form while enabling interactive functionality without structural compromise. Post-fabrication inspection confirmed dimensional precision and mechanical robustness, indicating suitability for repeated classroom use. Overall, the fabricated model represents the successful transition from digital design to a tangible, interactive learning tool, effectively bridging theoretical instruction with hands-on experiential learning, particularly for visually impaired users who rely on tactile feedback.

### Final assembled reflexology teaching robot

**Fig. 6:**
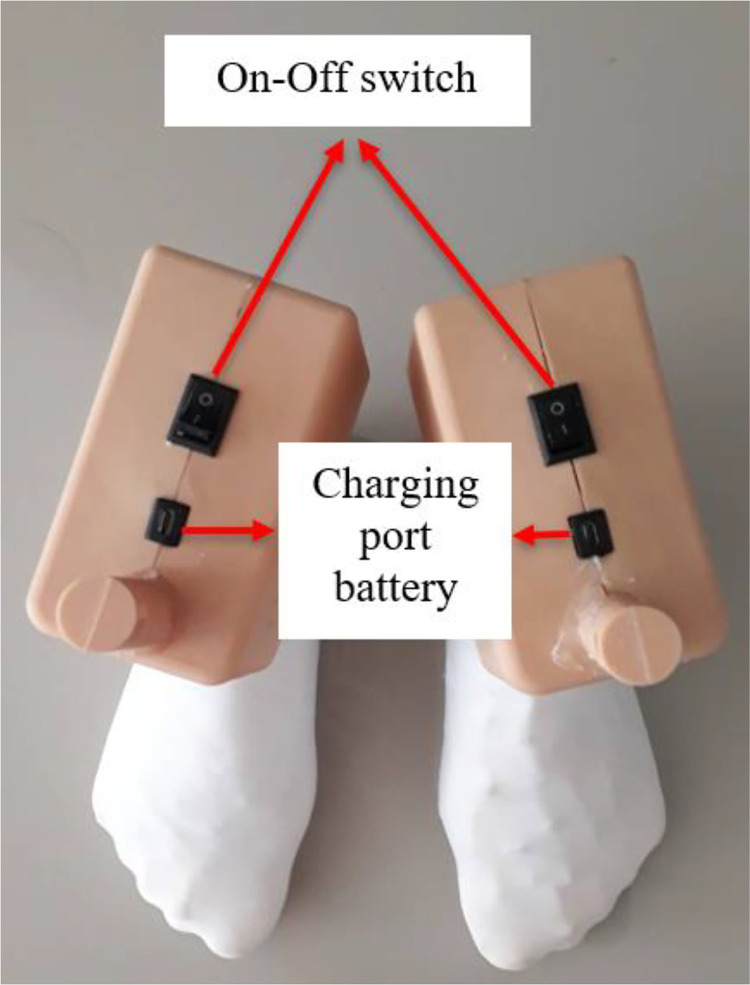
Presents fully assembled foot reflexology teaching robot for instructional.

The final assembly stage, shown in Fig. 6, integrates the 3D-printed foot enclosure with the control circuit, tactile sensors, internal battery, and user interface components, marking the transition from prototype construction to a fully operational educational device. The additively manufactured casing securely houses all electrical elements while preserving the anthropomorphic geometry required for accurate reflexology instruction. Ergonomically positioned components, including the On–Off switch and battery charging port, enhance accessibility and operational efficiency for both learners and instructors. The assembly process was guided by considerations of maintainability, portability, and structural durability to support repeated classroom or laboratory use. Modular electrical connections facilitate simplified maintenance, battery replacement, and charging, supporting long-term usability and sustainability. Each robotic foot unit operates independently, enabling flexible instructional configurations and precise demonstration of reflexology zones and pressure application. Overall, the completed system demonstrates the effective integration of mechanical, electrical, and ergonomic design principles through additive manufacturing, resulting in a durable, portable, cost-effective, and accessible assistive learning device tailored to support learners with visual impairments.

### Supporting software and equipment

**Fig. 7:**
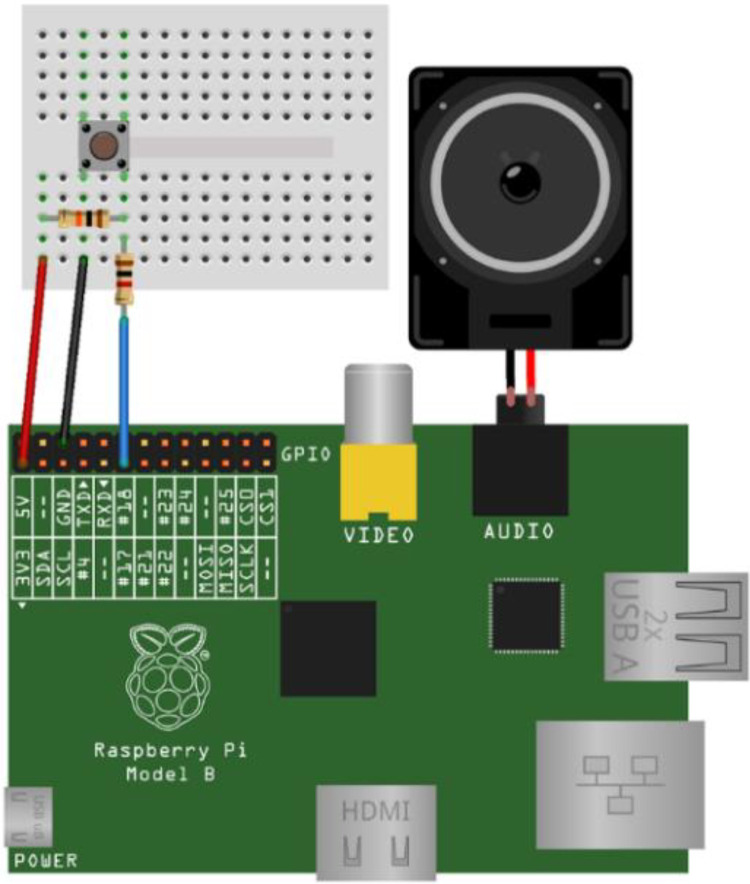
Configuration and interconnection of the operational circuit components.

Signals from the individual switches are transmitted to the control board, which processes the corresponding input commands. Upon activation of a switch, a pre-recorded audio file is triggered to deliver spoken instructional guidance. The fundamental circuit configuration is illustrated in Fig. 7.

**Fig. 8:**
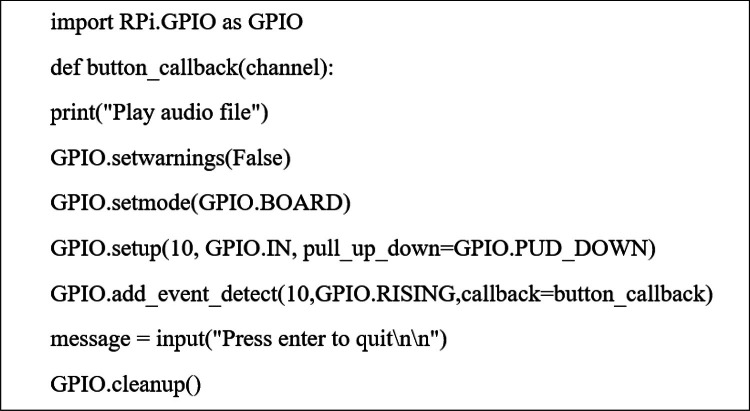
Presents the source code utilised in the programming implementation.

The Raspberry Pi board was programmed using Python, a cross-platform programming language widely supported across Unix, Linux, Windows, and other operating systems. As an open-source platform, Python enables cost-free development and continuous community-driven enhancement, making it well suited for embedded and educational applications. In this study, Python was employed to develop a control program for audio playback associated with instructional media. In the initial implementation, the system was configured to detect switch activation and execute a command that prints the message “Play audio file.” An example of the Python code is presented in Fig. 8.

### Evaluation session with visually impaired learners

**Fig. 9:**
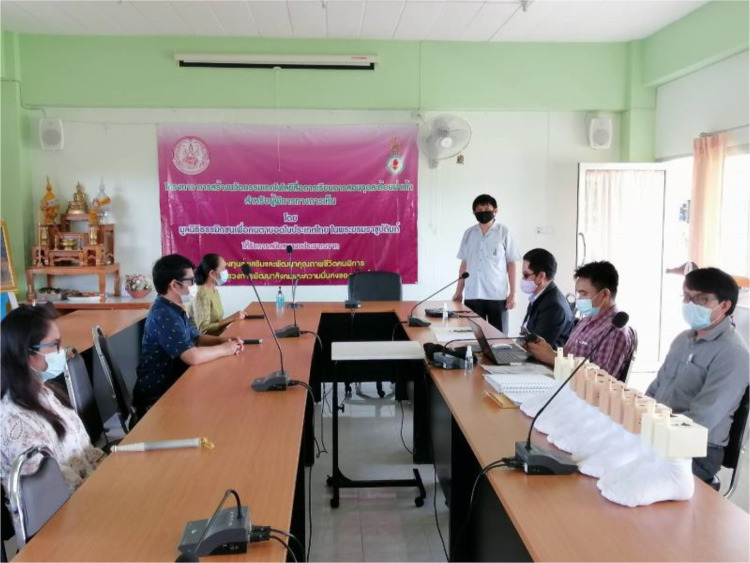
Evaluation of foot reflexology teaching robot during practical user interaction.

The evaluation setup is shown in Fig. 9. Fig. 9 presents a structured evaluation session of the foot reflexology teaching robot, demonstrating its implementation within a controlled educational setting. Structured validation sessions were conducted with five visually impaired adult participants and two instructors in a controlled educational environment. Visually impaired learners, supported by facilitators, are arranged around a conference-style table and interact with the robotic system through guided instruction, engaging in guided tactile exploration while receiving real-time auditory feedback generated by the system, enabling hands-on, experiential learning without reliance on visual cues. This evaluation session enables systematic assessment of learner performance, haptic feedback perception, and overall usability, while allowing instructors to observe, record, and analyse skill development in a structured manner. Furthermore, the activity illustrates the robot’s value as a multisensory pedagogical tool, providing guided practice while generating evidence to inform iterative design improvements. Overall, the evaluation confirmed the system’s ability to deliver effective multisensory, non-visual learning experiences and provided critical data to inform iterative design refinement. This **Method validation**

### Technical validation

Sensor calibration confirmed uniform response accuracy across all 12 predefined reflex zones, demonstrating reliable tactile activation and signal transmission. The average system activation delay was measured at 0.28 ± 0.04 s, ensuring real-time feedback suitable for instructional use. Audio output levels exceeded 85 dB under standard classroom conditions, providing clear and accessible auditory cues for visually impaired users. Mechanical inspection conducted after two hours of continuous operation revealed no observable dimensional deformation or structural degradation, validating the dimensional stability and durability of the PLA-based 3D-printed structure. In addition, the integrated power management circuit supported up to three hours of autonomous operation per charge, confirming suitability for extended teaching sessions.

The fabrication and assembly stages confirmed the effectiveness of the design-to-prototype workflow. PLA-based additive manufacturing enabled the production of a lightweight, cost-effective, and dimensionally stable prototype suitable for repeated educational use. The final assembly incorporated essential operational features, including switch-controlled inputs, an internal battery charging system, and modular electrical connections, enhancing maintainability, portability, and long-term usability. Integration of a Raspberry Pi control platform with Python-based programming provided responsive audio guidance linked to tactile interaction, establishing an interactive multisensory learning tool.

### Educational validation

As illustrated in Fig. 7, visually impaired learners were able to effectively identify and activate reflexology zones through combined tactile exploration and auditory confirmation. Observational analysis indicated that participants could independently locate and correctly activate approximately 80 % of reflex points following two guided sessions of 20 min each. Qualitative feedback from both learners and instructors highlighted increased confidence, engagement, and independence in acquiring reflexology skills, demonstrating the effectiveness of multisensory, non-visual learning interactions enabled by the system. The survey investigated the integration of teaching and learning among 29 visually impaired participants across multiple study locations at Khon Kaen Vocational College for the Blind. The results were systematically analyzed using established statistical methods. Descriptive statistics, including the mean and standard deviation, were employed to evaluate and summarize survey responses.

The assessment findings indicate that the security dimension achieved the highest mean score (*M* = 3.8621, SD = 0.3509), reflecting strong user confidence in system safety. Practicality was also rated highly (*M* = 3.8276, SD = 0.4682), followed by convenience and ease of use (*M* = 3.7586, SD = 0.4355) and durability (*M* = 3.7586, SD = 0.4355). Efficiency received a slightly lower, yet favourable, evaluation (*M* = 3.7241, SD = 0.6490), indicating some variability in perceived performance. Overall, the combined results yielded a mean score of 3.7862 (SD = 0.0567), indicating a consistently positive assessment across all evaluated dimensions. The relatively low overall standard deviation reflects strong agreement among participants and confirms the reliability and effectiveness of the system in meeting user expectations. Collectively, these findings confirm that the proposed reflexology teaching robot represents a viable, accessible, and sustainable educational innovation developed through multidisciplinary design and rapid prototyping.

### Validation outcome

The validation results confirm that the proposed method is functionally stable, repeatable, and user-inclusive. The integration of anatomically accurate 3D-printed geometry, sensor-based feedback mechanisms, and open-source control architecture enables straightforward replication using standard FDM 3D printers and commonly available microcontrollers. This validated workflow provides a scalable and cost-effective methodological template for the development of future assistive educational devices, particularly for learners with visual impairments. Implementation within a controlled educational environment further demonstrated the system’s pedagogical relevance. Structured evaluation sessions involving visually impaired learners confirmed the robot’s capacity to support experiential learning, precise identification of reflexology zones, and guided skill development. Observations of user interaction highlighted the effectiveness of haptic feedback and modular design in facilitating engagement and comprehension while providing a basis for iterative design improvement. structured assessment approach supports methodological reproducibility and demonstrates alignment with user-centred design principles for assistive educational technologies targeting learners with visual impairments.

## Limitations

Not applicable.

## Ethics statements

None.

## CRediT author statement

**Kriengkrai Nabudda**: Conceptualization, Investigation, Methodology, Software, Writing - Original Draft. **Kanokpit Nabudda**: Validation, Data curation, Investigation, Resources. **Nustha Kitprathaung**: Visualization, Investigation, Data Curation. **Suwilai Phumpho**: Conceptualization, Software, Methodology, Visualization. **Pongthep Poungthong**: Methodology, Formal analysis, Supervision, Project administration, Writing- Reviewing and Editing. **Arus Kongrungchok**: Validation, Writing- Reviewing and Editing, Supervision, Project administration.

## Supplementary material and/or additional information [OPTIONAL]

None.

## Related research article

None.

## For a published article

None.

## Declaration of competing interest

The authors declare that they have no known competing financial interests or personal relationships that could have appeared to influence the work reported in this paper.

## Data Availability

Data will be made available on request.
